# Diagnosis and treatment outcomes of primary lymphoma of the thyroid gland

**DOI:** 10.1007/s12672-025-03218-3

**Published:** 2025-08-16

**Authors:** Omar Hamdy, Gehad A. Saleh, Ramadan Ayman Selim, Radwa M. Abdelsattar, Maryam M. Dawood, Youssef Y. Abbas, Rana Abdo, Yousef M. Mansour, Jana M. AbuZahra, Mohamed Elsayed, Ahmed E. Eladl, Shaimaa El-Ashwah, Mohamed Ezat

**Affiliations:** 1 Surgical Oncology Department, Oncology Center, Mansoura University, Mansoura, Egypt; 2 Diagnostic and interventional radiology department, faculty of medicine, Mansoura University, Mansoura, Egypt; 3 Mansoura University Hospitals, Mansoura, Egypt; 4 Faculty of Medicine, Mansoura University, Mansoura, Egypt; 5 Endocrinology and diabetes unit, Internal Medicine Department, faculty of Medicine, Mansoura University, Mansoura, Egypt; 6 Pathology department, faculty of medicine, Mansoura University, Mansoura, Egypt; 7 Hematology unit, Oncology Center, Mansoura University, Mansoura, Egypt

**Keywords:** Thyroid lymphoma, Chemotherapy, Thyroid tumors, Thyroidectomy

## Abstract

**Introduction:**

Primary thyroid lymphoma (PTL) is a rare extranodal malignancy accounting for less than 5% of thyroid cancer, with a unique algorithm of management in contrast to other types of thyroid cancer, where surgery is the cornerstone of management. PTL usually shows female predominance, and diffuse large B-cell lymphoma (DLBCL) is the most prevalent pathological subtype. In this study, we aimed to assess thyroid lymphoma’s epidemiology, patterns of relapse, diagnostic approaches, and prognostic features, presenting a 16.5-year experience at a tertiary referral oncology center.

**Methods:**

This retrospective study assessed PTL patients managed between January 2008 and June 2024 at a tertiary referral center. All the clinical, diagnostic, therapeutic, and follow-up data were analyzed.

**Results:**

Sixteen patients were included, with a mean age of 59.34 years and a female-to-male ratio of 1.7. Most cases (68.8%) were diagnosed at stage II, with DLBCL identified in 93.8% of patients. Remission was achieved in half of the patients after first-line therapy, with no cures following second-line treatments. All the patients who died were stage II at the time of diagnosis. The median disease-free survival (DFS) was 35 months, and the overall survival (OS) was 37.5 months.

**Conclusion:**

Being aware of PTL can facilitate the timely initiation of treatment. Chemotherapy is the definitive treatment, used alone or with surgery, if done for diagnostic purposes. PTL has a good prognosis, however, late-stage disease can be linked to worse outcomes. This study underscores the importance of multimodal diagnostic and therapeutic strategies while highlighting the need for further research into this rare malignancy.

## Introduction

Primary thyroid lymphoma (PTL) is a unique and uncommon extra-nodal lymphoma that starts inside the thyroid gland. It is responsible for less than 5% of all thyroid cancers and 2% of extra-nodal lymphomas [1]. It is more common in women who frequently have a history of autoimmune thyroid diseases like Hashimoto’s thyroiditis, and primarily affects older adults [1]. The patient usually presents with an enlarged thyroid, causing compression and obstructive symptoms, and symptoms of hypothyroidism [2].

The majority of PTLs are derived from B-cells, and diffuse large B-cell lymphoma (DLBCL) is the commonest type [3], which makes up more than 50% of PTL cases. In comparison, mucosa-associated lymphoid tissue lymphomas (MALT) make up 20–30%. Follicular lymphoma accounts for 12%, while Hodgkin’s disease accounts for 7% of cases, small lymphocytic lymphoma for 4%, and Burkitt’s lymphoma for 4% of cases [1].

A PTL diagnosis is made using various techniques, such as fine needle aspiration biopsy (FNAB), core needle biopsy, incisional, and excisional biopsy, all of which use immunohistochemistry (IHC) and flow cytometry. PTL has also been accidentally discovered in resected thyroid specimens. Due to the rarity of PTL, there is little data to support the precision and dependability of diagnostic techniques [4]. Flow cytometry was reported to provide an additional tool that can lead to the establishment of the diagnosis [5].

There are no specific laboratory or imaging features for the accurate diagnosis of PTL. It is usually associated with high TSH and low T3 and T4, while on ultrasonography (US), it shows high vascularity and multiple hyperechogenic areas in diffuse, nodular, or mixed patterns [6, 7]. The treatment of PTL varies according to subtype. It includes a multimodal strategy based mainly on chemotherapy and radiotherapy, while surgical intervention is reserved as a last diagnostic step [8, 9].

In this study, we aimed to assess thyroid lymphoma’s epidemiology, patterns of relapse, diagnostic approaches, and prognostic features, presenting a 16.5-year experience at a tertiary referral oncology center.

## Materials and methods

### Study population

The local institutional review board approved this retrospective study under R.24.12.2962. The institutional registry at our center was searched for pathologically proven thyroid lymphoma patients who were followed up between January 2008 and June 2024. We excluded patients with missing or non-registered data.

### Clinical and laboratory data

For all the included patients, the data was retrieved from the prospectively maintained hospital electronic database including epidemiological data (age, gender, body mass index (BMI), medical comorbidities), clinical data, basic laboratory tests (complete blood count and thyroid function), and prognostic data (lymphoma types, stage, treatment, relapse, and survival).

### Imaging

#### Sonographic examination

US was performed for all patients, using a high-resolution phased-array linear transducer to assess the thyroid lesions and the cervical lymph nodes (LNs).

#### Computed tomography (CT) technique

**Fig. 1:**
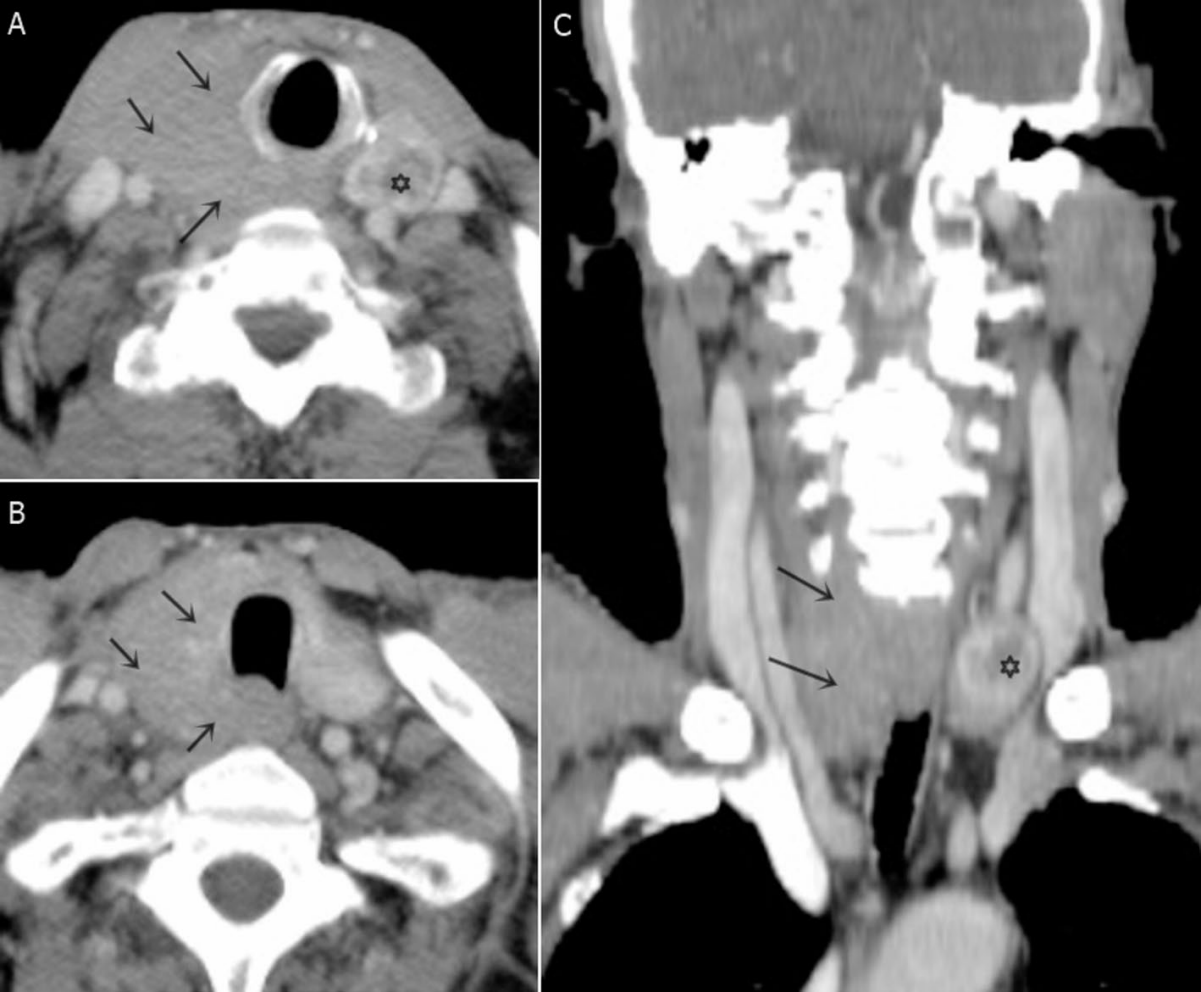
CT scan of a case of PTL: A 66-year-old male with non-Hodgkin lymphoma (DLBC type) in the right thyroid lobe. Post-contrast CT neck axial (**A & B**) and reconstructed coronal (**C**) images revealed infiltrative hypo-enhancing right thyroid lobe mass (arrows) measures 5.5 × 3 × 9 cm, it’s seen inseparable from Rt sternomastoid muscle laterally, contacting both right common carotid artery (CCA), right internal jugular vein (IJV) without caliber changes and extending into post cricoid region posteromedially. No detected tracheal deviation. A small left thyroid hypodense nodule (asterisk) measures 1 cm. No detected suspicious cervical LNs

CT scans (Fig. 1) were performed for all patients on a 64 multidetector CT scanner (Phillips Medical Systems, Best, The Netherlands) or a 128 multidetector CT scanner (GE Revolution). Patients were examined in a supine position, craniocaudal direction, scanning started from the level of the skull base to the upper mediastinum. For contrast-enhanced CT, an automated injector administered a bolus dose of 90 ml of iodinated non-ionic contrast agent (Ultravist 300) intravenously at a rate of 2.5-3 ml/s. Sagittal and coronal reconstruction images were obtained for all patients with a reconstruction slice thickness of 2.5 mm.

#### Image interpretation

Image analysis was performed by a consultant radiologist for the following features for each thyroid lesion: [1] the size (the maximum diameter in cm) [2] the location (right lobe, left lobe, or bilateral); [3] the number (single, multiple); [4] US features and TIRADS category (composition, echogenicity, shape, margin); [5] CT features (enhancement, necrosis, calcification, tracheal deviation, invasion of the surrounding tissue); [6] presence of suspicious cervical LNs (globular or rounded shape, enlarged with short axis ≥ 10 mm, cystic changes, heterogenous echogenicity or enhancement).

#### 18 F-FDG PET/CT technique

**Fig. 2:**
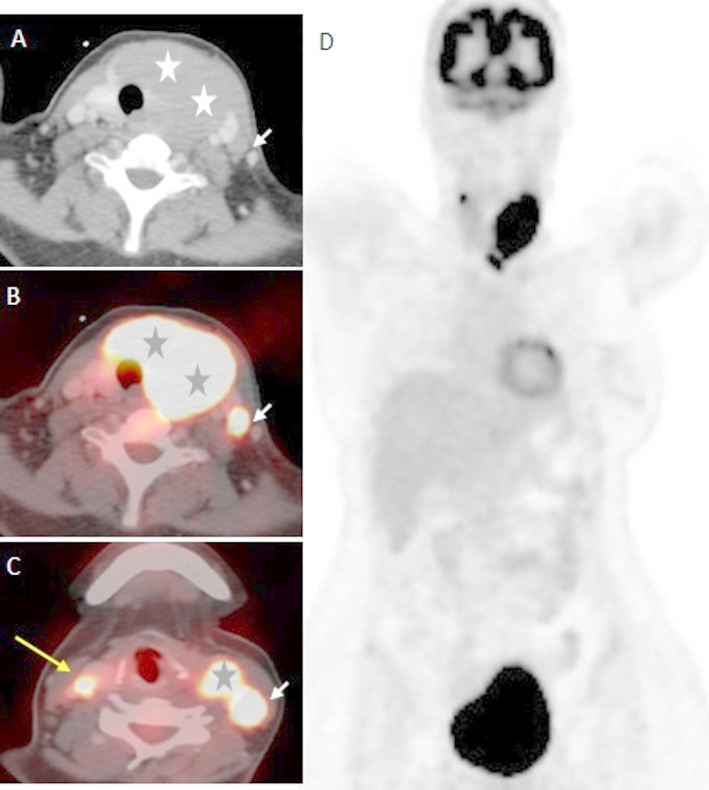
Baseline PET-CT of a patient PTL Axial post-contrast CT (**A**) and axial fused PET-CT (**B&C**) revealed metabolically avid diffuse left thyroid and isthmic lesion (stars) displacing left CCA and internal jugular vein laterally without vascular invasion (SUVmax 25). Small right thyroid lobe nodule with similar metabolic activity (long arrow). Few left level III cervical LNs, the largest of SUV max about 21 (short arrows). Coronal reformatted maximum intensity projection (MIP) image (**D**) confirms the absence of other metabolically active lesions

**Fig. 3:**
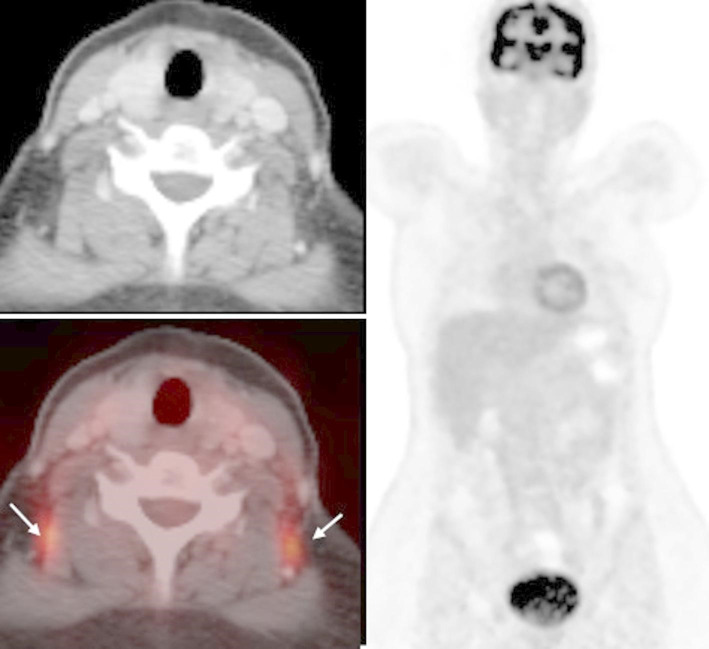
Follow-up PET-CT of the same patient with PTL Axial post-contrast CT (**A**), axial fused PET-CT (**B**) and coronal reformatted MIP image (**C**): complete metabolic remission mad morphologic resolution of all thyroid lesions and left cervical LNs with restoration of normal CT appearance and size of left thyroid lobe. Symmetrical intense FDG uptake in the paravertebral regions (short arrows), corresponding to fat areas without CT abnormalities compatible with activated brown adipose fat

Whole body 18 F-FDG positron emission tomography/computed tomography (18 F-FDG PET/CT) was performed in nine patients when histopathological diagnosis of lymphoma was confirmed. A whole-body PET study was done from the skull to the mid-thighs with a scan time of about 30–45 min. A diagnostic enhanced whole-body CT scan was performed using an automated injector with a 125 ml adult dose of iodinated contrast media at a rate of 4 ml/sec. **(**Figs. 2 and 3).

#### Image interpretation

Cross-linking of axial, coronal, and sagittal reformats for PET, CT, and combined CT images was performed. The SUV mean of the liver and mediastinal pool was measured. Abnormal tracer uptake was judged visually and semi-quantitatively by driving a region of interest (ROI) in the metabolically active lesions and measuring SUVmax.

### Pathological examination

The microscopic examination of the tumor -whether by an FNAB, core needle, or surgical biopsy- was performed by expert pathologists. The Hematoxylin and Eosin-stained slides were initially examined. Then the diagnosis was confirmed by the immunohistochemical (IHC) studies as recommended for each subtype.

### Statistical analysis

The data was analyzed using the statistical package of social sciences (SPSS 25, IBM/SPSS Inc., Chicago, IL), including descriptive and analytical statistics. Descriptive statistics comprised estimations for describing continuous data as mean (X) and standard deviation (SD) for normally distributed data, or median (Med) and range for skewed data. For quantitative data, frequency was expressed as a percentage (%). DFS was measured for the patients who completed their follow-up, in months, from the date of complete response to the date of death, relapse, or the last follow-up visit. OS was measured for the patients who completed their follow-up, in months, from the date of initial diagnosis to the date of death or the last follow-up visit.

## Results

### Epidemiology

Sixteen patients diagnosed with PTL were included. The mean age of participants was 59.34 ± 13.6 years. Among them, 62.5% (10 patients) were females, and 37.5% (6 patients) were males. Neck swelling alone was the most frequent presenting complaint in 75% of the cases or in association with other complaints (18.8%). Thyroid function was normal in eleven patients, hypothyroid in four patients, and unrecorded in one patient. Multifocal tumors were observed radiologically in eight patients, and the neck LNs were not suspicious in 12 (75%). The demographic and diagnostic characteristics are summarized in Table 1.

**Table 1:** The demographic, clinical, and radiological data of the included patients

Variables	Study cases *n* = 16	
	Number	Percent
**Sex**		
Male	6	37.5
Female	10	62.5
**Comorbidities**		
Diabetes Miletus	2	12.5
Hypertension	5	31.2
**Complaint**		
Inguinal swelling	1	6.2
Neck swelling	12	75
Neck swelling + dysphagia	1	6.2
Neck swelling + dyspnoea	1	6.2
Neck swelling + hoarseness of voice	1	6.2
**Site**		
Bilateral	8	50
Left	3	18.8
Right	4	25
N/A	1	6.2
**Focality**		
Multifocal	8	50
N/A	1	6.2
Unifocal	7	43.8
**TIRADS**		
4a	1	6.2
4b	2	12.5
4c	2	12.5
5	3	18.8
N/A	8	50
**Lymph nodes**		
No	12	75
Suspicious	4	25
**Laterality of lymph nodes**		
Bilateral	1	6.2
Ipsilateral	3	18.8
No	12	75
**Thyroid profile**		
Euthyroid	11	68.8
Hypothyroid	4	25
N/A	1	6.2

	Mean ± SD	Median (Range)
Age (years) (*n* = 16)	59.34 ± 13.6	
Radiological Size (cm) (*n* = 13)		6.5 (2.2–11)

Continuous data expressed as mean ± SD and median (range)

Categorical data are expressed as numbers (percent)

### Diagnosis

**Fig. 4:**
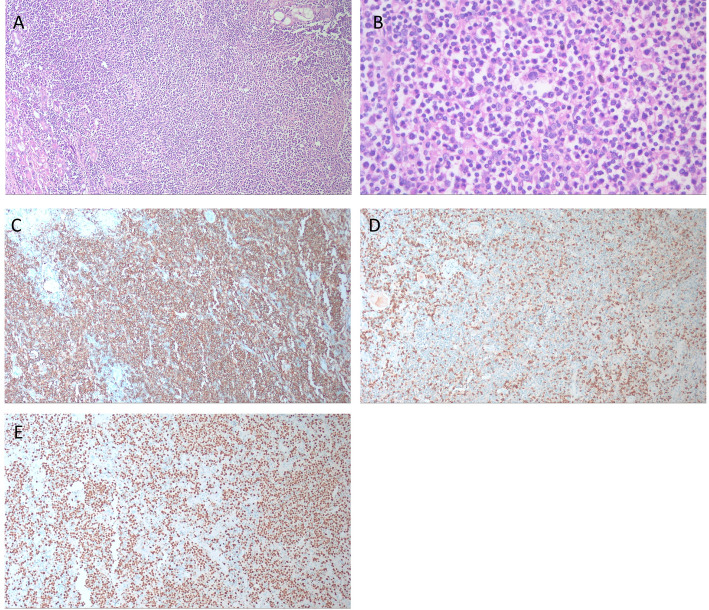
Microscopic examination of a case of PTL (**A**) Effacement of thyroid architecture by diffuse infiltration of large transformed lymphocytic cells. Noted remnants of small, atrophied thyroid follicles at the side. (**B**) Atypical lymphocytic cells with large hyperchromatic nuclei and scanty cytoplasm, exhibiting a moderate degree of atypia. (**C**) Diffuse positive CD20 IHC reaction highlighting the large, atypical B-cell lymphocytes. (**D**) Negative CD3 IHC reaction in the large atypical lymphocytic cells, with scattered positive small reactive T-cell lymphocytes in the background. (**E**) Ki-67 IHC showing a high proliferation index

Histopathological examination (Fig. 4) confirmed DLBCL in fifteen patients (93.8%) and MALT lymphoma in one patient. Staging revealed five patients in Stage I and eleven in Stage II. The Immunohistochemical (IHC) markers were evaluated for the study. A summary of the IHC results is presented in Table 2, where CD20 was positive and CD3 was negative in all the included cases. The pathological and staging data are summarized in Table 3.

**Table 2:** Results of the IHC staining used in the included samples

IHC stain type	Positive	Negative	*N*/A
CD 20	16 (100%)	0 (0%)	0 (0%)
CD 3	0 (0%)	13 (81.3%)	3 (18.8%)
CD 10	5 (31.3%)	3 (18.8%)	8 (50%)
CD 5	0 (0%)	3 (18.8%)	13 (81.3%)
CD 23	0 (0%)	1 (6.2%)	15 (93.8%)
CD 30	0 (0%)	4 (25%)	12 (75%)
CD 138	1 (6.2%)	0 (0%)	15 (93.8%)
BCL 2	6 (37.5%)	2 (12.5%)	8 (50%)
BCL 6	6 (37.5%)	1 (6.2%)	9 (56.3%)
MUM 1	3 (18.8%)	1 (6.2%)	12 (75%)
Cyclin D1	0 (0%)	2 (12.5%)	14 (87.5%)
CK 19	0 (0%)	1 (6.2%)	15 (93.8%)
Ki 67*	7 (34.8%)	0 (0%)	9 (56.3%)
Thyroglobulin	0 (0%)	3 (18.8%)	13 (81.3%)
TTF 1	0 (0%)	5 (31.3%)	11 (68.8%)
EMA	1 (6.2%)	1 (6.2%)	14 (87.5%)

IHC: Immunohistochemical

*Ki67: High or low (not positive or negative)

**Table 3:** The pathological & staging data of the included patients

Variables	Study cases *n*= 16	
	Number	Percent
**Preoperative FNAB (Bethesda)**		
1	2	12.5
2	5	31.2
3	2	12.5
4	1	6.2
5	1	6.2
N/A	2	12.5
NHL	3	18.8
**Final biopsy results**		
FNAB	3	18.8
Incisional	1	6.2
Thyroidectomy	9	56.3
Trucut	3	18.8
**Lymphoma subtype**		
DLBCL	15	93.8
MALT	1	6.2
**Stage**		
I	5	31.2
II	11	68.8
**Laterality**		
N/A	8	50
Bilateral	3	18.8
Unilateral	5	31.2
**Focality**		
N/A	8	50
Diffuse	1	6.2
Multifocal	4	25
Unifocal	3	18.8

	Mean ± SD	Median (Range)
Pathological size (cm) (*n* = 8)		7 (4.5–6)

Continuous data expressed as mean ± SD and median (range)

Categorical data are expressed as numbers (percent)

NHL: Non-Hodkin lymphoma, DLBCL: diffuse large B cell lymphoma, MALT: mucosa-associated lymphoid tissue, FNAC: fine needle aspiration cytology, N/A: not available

### Treatment and patterns of relapse

**Fig. 5:**
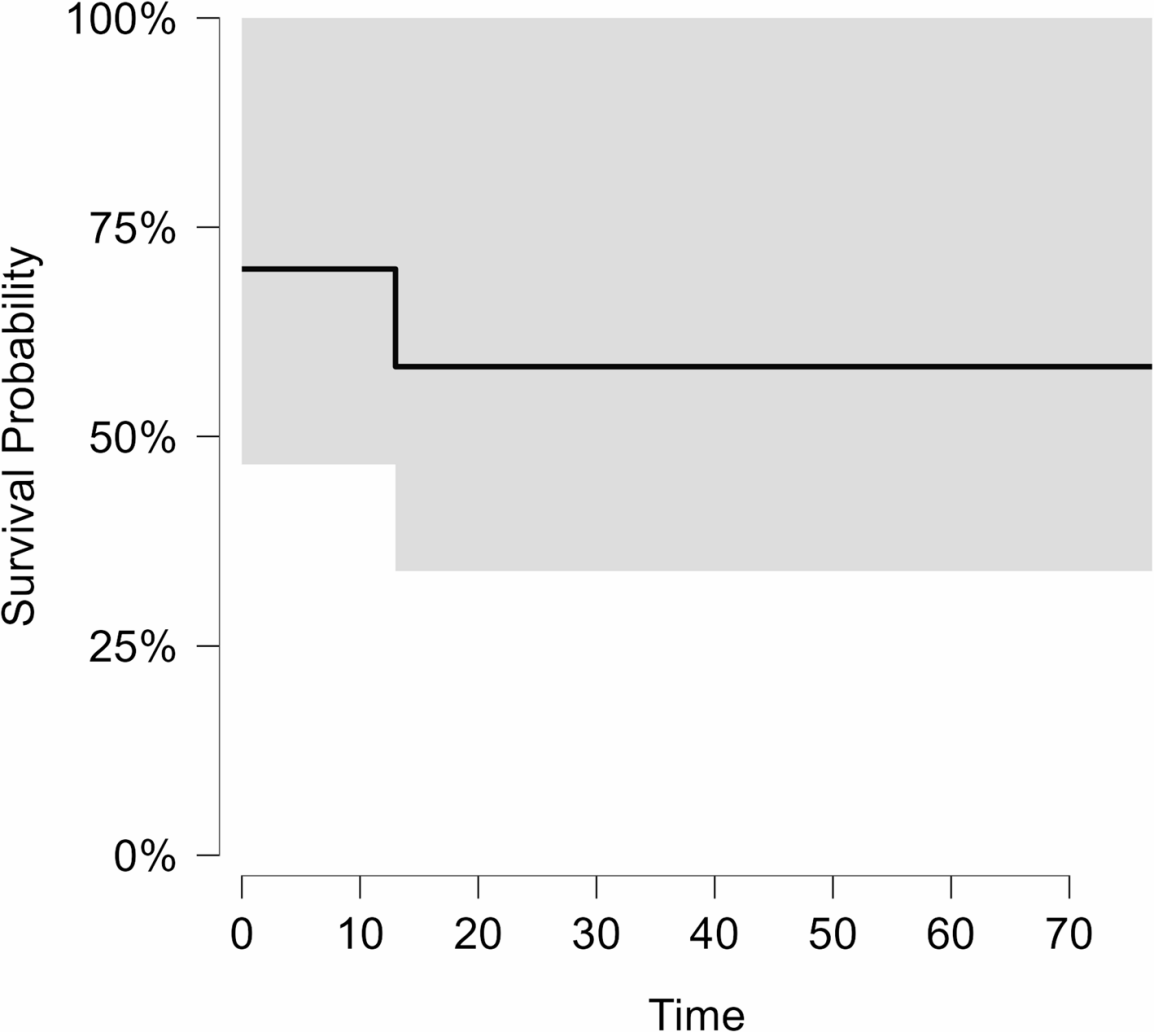
Kaplan-Meier curve of DFS

**Fig. 6:**
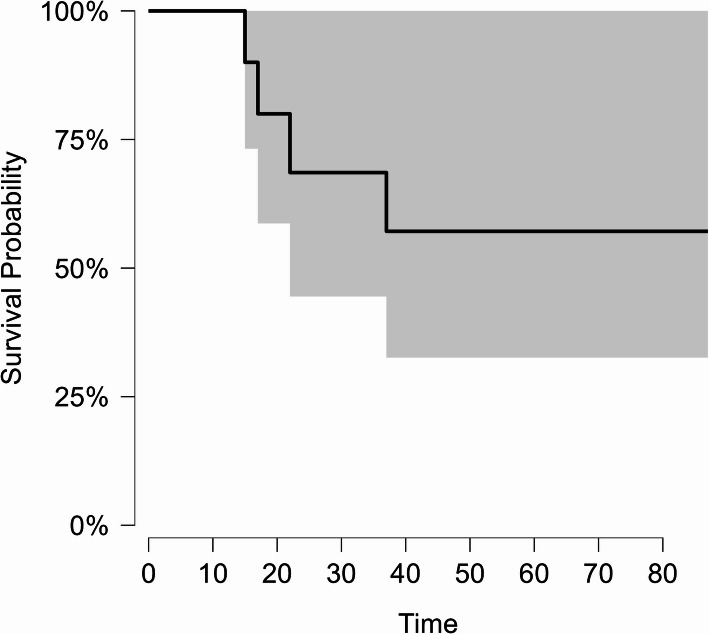
Kaplan-Meier curve of OS

Chemotherapy was the most common treatment modality, and it was used in fifteen cases (93.8%). It was used as definitive treatment in nine patients and in combination with surgery in six patients, while one patient who had the diagnosis of MALT lymphoma underwent surgery alone. Chemotherapy regimens included R-CHOP in eight patients, CHOP in five patients, COP, and mini-CHOP each in one patient. Eight patients achieved remission after the first-line chemotherapy, while four required second-line treatment protocols, which included DHAP and GDP due to resistance to first-line therapy. Two patients showed relapse in the form of generalized lymphadenopathy after initial remission. At the date of the last visit, six patients were alive, four patients had died, and six patients were lost to follow-up. All the patients who died were stage II at the time of diagnosis. The median DFS was 35 months (Fig. 5), and the OS was 37.5 months (Fig. 6). The treatment details and outcomes are summarized in Table 4.

**Table 4:** Treatment and oncological outcomes

Variables	Study cases *n* = 16		
	Number	Percent	
**Treatment**			
Chemotherapy	8	50.0	
Surgery	1	6.2	
Surgery + chemotherapy	7	43.8	
**Type of surgery**			
total thyroidectomy	8	50.0	
Tracheostomy	2	14.3	
**Chemotherapy protocol (n = 15)**			
CHOP	5	33.3	
COP	1	6.7	
Mini-CHOP	1	6.7	
R-CHOP	8	53.3	
Complete remission after the first line	8	50	
Second line treatment	4	25	
**Status at last visit**			
Alive	6	37.5	
Dead	4	25	
Lost follow up	6	37.5	

	Mean ± SD	Median (Range)	
Operative time (*n* = 8) (minutes)		127.5 (60–420)	

Continuous data expressed as mean ± SD and median (range)

Categorical data are expressed as numbers (percent)

## Discussion

PTL is more common in females than males, with a ratio of 1.6. This is remarkable, as other types of lymphomas are more prevalent in men [10]. The typical age upon diagnosis is between 50 and 80 years old, with the 7th and 8th decades being the highest. Rarely is PTL diagnosed in those under forty [11–13]. Similar to data from literature, in our study, the median age was 59.34 ± 13.6 years. As a known risk factor and possible precursor for PTL, Hashimoto’s thyroiditis could account for the higher age range and female preponderance of patients in comparison to other types of lymphoma [10, 14, 15]. In our cohort, the female-to-male ratio was 1.7.

The most common clinical presentation of PTL is progressive thyroid enlargement, sometimes in a brief period, with or without cervical lymphadenopathy, and compressive symptoms on the surrounding structures, such as vocal cord paresis, dysphagia, dyspnea, or dysphonia [16, 17]. In our cohort, only one patient presented with dyspnea and another one with dysphagia. Four patients were hypothyroid while eleven patients were euthyroid.

DLBCL is the most prevalent subtype of PTL, according to several earlier studies. Other common pathologies include follicular lymphoma and marginal zone, or MALT lymphoma [3, 18–20]. In our study, fifteen out of the included sixteen patients had DLBCL, while only one patient had MALT lymphoma. No cases of follicular or marginal zone lymphoma were recorded.

In terms of the diagnosis of PTL in our study, thyroid resection and open surgical biopsy were the most commonly used techniques. FNAB or core needle biopsy may offer a way to lessen the need for more invasive procedures and shorten the interval between clinical presentation and treatment when combined with flow cytometry, IHC, and fluorescence in situ hybridization (FISH) to check for gene rearrangement [4]. In our cohort, the majority of the included cases were diagnosed only after surgical intervention, while FNAB established the diagnosis only in three out of sixteen patients (18.75%). This agrees with the findings of the recently published multicenter study by Su M et al. [21]. All the included cases were positive for CD20, while markers such as CD10, BCL 2 & BCL 6 were expressed in 5,6, and 6 cases, respectively.

Nearly one-third of our patients (31.2%) were diagnosed as stage I, while the other two-thirds (68.8%) were diagnosed as stage II. In literature, stage I-IIE was the diagnosis for 88% of patients, according to Graff-Baker et al. [13]. Also, in a study by Chai et al., 92.1% of patients had a stage I–IIE diagnosis [14].

The management of PTL was based on many factors, including the histological type and the extent of the disease. The treatment consisted of chemotherapy, surgery, or a combination of both. Graff-Baker et al. discovered that 61% of patients in the Surveillance, Epidemiology, and End Results (SEER) database received surgical treatment, which is relatively high [13]. This is more than anticipated for a cancer where chemotherapy, with or without radiation, is the recommended course of treatment [13, 18, 22]. But it should be kept in mind that, on the contrary, surgical treatment alone is still advocated as a therapeutic option in stage IE tumors [23]. In our study, the most commonly used treatment modality was chemotherapy, with which fifteen patients were treated alone or in combination with diagnostic surgery. Four patients required second-line protocols due to the persistence of the disease despite initial treatment with first-line protocols.

PTL has had a good prognosis overall, with a high 5-year survival rate. Lack of treatment, advanced disease stage, follicular or diffuse large cell histological subtypes, and advanced age (over 80) are all linked to a worse prognosis [11, 12, 24]. Graff-Baker et al.‘s study included 1,408 patients from the SEER database and identified a median all-cause survival of 9.3 years and a 5-year overall survival of 66% [13]. A more recent analysis by Zhu Y et al. defined age, marital status, histological subtypes, stage, and treatment modalities as strong predictors of PTL-specific risk of death [25]. Regarding our results, remission was achieved in half of the patients after first-line chemotherapy, but no cures were reported after second-line treatments. At the end of the follow-up period, six patients were alive, four had died, and six were lost to follow-up. The median DFS was 35 months, and the OS was 37.5 months. This discrepancy of DFS & OS of the published studies can be attributed to three main factors: significantly shorter follow-up periods, the small sample size, and the discrepancies in the pathological variants.

It is acknowledged that this study has limitations. The most important one is the small number of patients. Additionally, it was only a single-center retrospective study, and we lost follow-up with some patients. Also, only nine out of sixteen patients underwent a PET-CT at the time of initial diagnosis. But it has strengths also; it presents a 16.5-year experience in a tertiary referral center of such a cohort. It also presents a rare form of thyroid malignancy that needs deeper understanding and research.

## Conclusion

Thyroid lymphoma is a unique entity of thyroid neoplasms that is usually present as thyroid tumors with neck swelling, not with systemic symptoms, but treated as lymphoma with chemotherapy, not with surgery. Surgery is reserved for diagnostic purposes when minimally invasive techniques are not sufficient to establish the diagnosis. IHC plays a crucial role in its diagnosis. Despite the rule that it carries a favorable prognosis, delayed stages at presentation can be linked to poor survival outcomes.

## Data Availability

All the clinical, radiological & pathological data used in this manuscript are available on the Mansoura University medical system (Ibn Sina Hospital management system). http://srv137.mans.edu.eg/mus/newSystem/.
